# Intercellular Transmission of a Synthetic Bacterial Cytotoxic Prion-Like Protein in Mammalian Cells

**DOI:** 10.1128/mBio.02937-19

**Published:** 2020-04-14

**Authors:** Aida Revilla-García, Cristina Fernández, María Moreno-del Álamo, Vivian de los Ríos, Ina M. Vorberg, Rafael Giraldo

**Affiliations:** a Department of Cellular and Molecular Biology, Centro de Investigaciones Biológicas (CIB-CSIC), Madrid, Spain; b Proteomics Facility, Centro de Investigaciones Biológicas (CIB-CSIC), Madrid, Spain; c German Center for Neurodegenerative Diseases (DZNE), Bonn, Germany; University of Edinburgh

**Keywords:** bacterial amyloidosis, prion-like, RepA-WH1, intercellular transmission, proteotoxic cross-aggregation

## Abstract

RepA is a bacterial protein that builds intracellular amyloid oligomers acting as inhibitory complexes of plasmid DNA replication. When carrying a mutation enhancing its amyloidogenesis (A31V), the N-terminal domain (WH1) generates cytosolic amyloid particles that are inheritable within a bacterial lineage. Such amyloids trigger in bacteria a lethal cascade reminiscent of mitochondrial impairment in human cells affected by neurodegeneration. To fulfill all the criteria to qualify as a prion-like protein, horizontal (intercellular) transmissibility remains to be demonstrated for RepA-WH1. Since this is experimentally intractable in bacteria, here we transiently expressed in a murine neuroblastoma cell line the soluble, barely cytotoxic RepA-WH1 wild type [RepA-WH1(WT)] and assayed its response to exposure to *in vitro*-assembled RepA-WH1(A31V) amyloid fibers. In parallel, murine cells releasing RepA-WH1(A31V) aggregates were cocultured with human neuroblastoma cells expressing RepA-WH1(WT). Both the assembled fibers and donor-derived RepA-WH1(A31V) aggregates induced, in the cytosol of recipient cells, the formation of cytotoxic amyloid particles. Mass spectrometry analyses of the proteomes of both types of injured cells pointed to alterations in mitochondria, protein quality triage, signaling, and intracellular traffic. Thus, a synthetic prion-like protein can be propagated to, and become cytotoxic to, cells of organisms placed at such distant branches of the tree of life as bacteria and mammalia, suggesting that mechanisms of protein aggregate spreading and toxicity follow default pathways.

## Abstract (precis)

Proteotoxic amyloid seeds can be transmitted between mammalian cells, arguing that the intercellular exchange of prion-like protein aggregates can be a common phenomenon. RepA-WH1 is derived from a bacterial intracellular functional amyloid protein, engineered to become cytotoxic in Escherichia coli. Here, we have studied if such bacterial aggregates can also be transmitted to, and become cytotoxic to, mammalian cells. We demonstrate that RepA-WH1 is capable of entering naive cells, thereby inducing the cytotoxic aggregation of a soluble RepA-WH1 variant expressed in the cytosol, following the same trend that had been described in bacteria. These findings highlight the universality of one of the central principles underlying prion biology: No matter the biological origin of a given prion-like protein, it can be transmitted to a phylogenetically unrelated recipient cell, provided that the latter expresses a soluble protein onto which the incoming protein can readily template its amyloid conformation.

## INTRODUCTION

A hallmark of protein aggregates accumulating in human neurodegeneration is their ability to template the conformational change of soluble native protein molecules into highly ordered polymers with a cross-β structure termed amyloid (1, 2). This has been extensively characterized for the PrP prion in spongiform proteinopathies (3, 4), the amyloid beta (Aβ) peptides (5–7), and Tau (8–11) in Alzheimer’s and related dementias or for α-synuclein in Parkinson’s disease (12–15). Protein aggregates are cell-to-cell transmissible through either tunneling nanotubes or secretion as naked aggregates or when packaged into extracellular vesicles. These mechanisms underlie the stereotypical spreading of protein aggregates throughout connected brain regions (16–20). Such “horizontal” transmissibility, which partially resembles the “infectivity” of the PrP prion, qualifies Aβ peptides, Tau, and α-synuclein as prion-like proteins (or prionoids) (21, 22).

Intercellular aggregate transmission is not solely a characteristic of disease-associated proteins. Several proteins of lower eukaryotes have the ability to fold into self-templating protein aggregates that faithfully propagate in progeny and transmit to other cells during mating (23). Interestingly, the prion-like behavior is not restricted to the original host. For example, cell-to-cell propagation of amyloid aggregates has been successfully reported in mammalian cells for the NM prion domain of the Saccharomyces cerevisiae translation termination factor Sup35 (24–28). Sup35-NM can also propagate in bacteria, provided that a second specific prion-inducing amyloid required for the prionization of Sup35 in S. cerevisiae is also expressed in the recipient cells (29). The other way around, both the amyloidogenic sequence stretch in RepA-WH1 (30) and the prion domain in CbRho (31) can functionally replace Sup35 prionogenic sequences in a stop-codon read-through translation assay in yeast. The extracellular bacterial functional amyloid curli/CsgA can experimentally induce the aggregation of proteins involved in human amyloidosis (32–35). Interest in the interplay between bacterial and mammalian amyloids is now boosted because of the probable role of amyloids and metabolites from gut microbiota in triggering neuroinflammation (36, 37). However, the transmission of a bacterial prion, or a prion-like protein, that is cytotoxic *per se* to mammalian cells has not been reported yet. Such a report would demonstrate that a protein aggregate with no sequence similarity to any mammalian proteins is transmissible, arguing that, independently of the amino acid sequence, perhaps any proteinaceous aggregation seed can be transmitted between mammalian cells.

The bacterial prion-like protein RepA-WH1 represents a synthetic model of amyloid disease built on RepA, a protein that controls plasmid DNA replication through the assembly of functional amyloid oligomers that hamper premature rounds of origin firing (38, 39). RepA forms stable dimers in solution through its N-terminal WH1 domain, while the C-terminal WH2 domain provides the major DNA binding interface. Upon allosteric binding to distinct natural ligands (specific double-stranded DNA [dsDNA] sequences, acidic phospholipids) (40–42), RepA-WH1 dimers dissociate into metastable monomers that subsequently assemble as amyloid oligomers and fibers *in vitro* (43, 44). When expressed in Escherichia coli, the hyperamyloidogenic variant RepA-WH1(A31V) forms particles of various sizes, distributed across the bacterial cytosol (45). These particles are transmissible vertically (i.e., from mother to daughter cells) as two distinct aggregate strains with remarkable appearances and phenotypes: elongated and mildly cytotoxic and compact and acutely cytotoxic, respectively (46). Coexpression of soluble and aggregation-prone RepA-WH1 variants in E. coli demonstrated that the A31V variant can template its conformation on the parental wild-type (WT) protein (47). Systems analyses (48), together with *in vitro* reconstruction in cytomimetic lipid vesicles (42, 49), have suggested that RepA-WH1(A31V) oligomers target the internal bacterial membrane, hampering proton motive force and thus ATP synthesis and transport through membranes, and enhance oxidative stress. In parallel, protein factors mounting the defense against stress and envelope damage coaggregate with RepA-WH1(A31V) amyloids (48). Taking the data together, bacterial viability is severely compromised by RepA-WH1 amyloidosis, in a manner resembling that seen with some of the central mitochondrial routes found in human amyloidosis (50–53). However, E. coli is not suitable for addressing the issues of cell-to-cell transmissibility of protein aggregates and the subsequent intracellular amyloid cross-aggregation, since this Gram-negative bacterium does not take up large protein particles due to the insurmountable obstacle of its three-layered cell envelope.

To explore the ability of the prion-like protein RepA-WH1 to propagate in a heterologous host, here we exposed murine neuroblastoma cells, transiently expressing mCherry-tagged soluble RepA-WH1(WT), to *in vitro*-assembled RepA-WH1(A31V) amyloid fibers. In addition, we studied the intercellular induction of protein aggregation by coculturing murine cells releasing RepA-WH1(A31V) aggregates with human neuroblastoma cells stably expressing soluble RepA-WH1(WT). Confocal microscopy and biochemical studies showed that the mammalian cells were able to take up RepA-WH1(A31V) amyloid fibers, which cross-seed the cytosolic aggregation of the endogenous RepA-WH1(WT) in the recipient cells. Moreover, coculture of cells releasing the RepA-WH1(A31V) variant also induced the aggregation of RepA-WH1(WT) in bystander cells, suggesting intercellular transmission of RepA-WH1(A31V) seeds. In both setups of experimental transmission, the induced RepA-WH1(WT) aggregates were cytotoxic and amyloidogenic in nature, as indicated by cell viability assays and by their affinity for a fluorescent compound specific for amyloids, respectively. The observed toxicity was dependent on the expression of RepA-WH1(WT) in the recipient cells, suggesting that it is linked to the intracellular assembly of aggregates of this protein and not simply to the uptake of toxic polymers. This confirmed that RepA-WH1, whose sequence shows no similarity matches with the human proteome, is a biosafe prion-like protein. The analyses of the proteomes of either murine cells exposed to the *in vitro*-assembled fibers or human cells internalizing the extracellularly released aggregates point to the impairment of mitochondria and intracellular trafficking and protein quality control networks in RepA-WH1 toxicity. The results presented here validate RepA-WH1 as a cytotoxic bacterial prion-like protein.

## RESULTS

### Transient expression in mammalian cells of variants of the RepA-WH1 protein recapitulates their aggregation phenotypes in bacteria.

Previous findings on the synthetic amyloid proteinopathy elicited in bacteria by the prion-like protein RepA-WH1 suggested that it might have features in common with a wide spectrum of human neurodegenerative amyloidoses. To assess if the model protein RepA-WH1, and its variants exhibiting distinct aggregation propensities in bacteria (45–47), would show a comparable behavior in mammalian cells, proteins were transiently expressed in murine N2a and human SH-SY5Y neuroblastoma cell lines, commonly used in studies of amyloid neurodegeneration, as well as in nonneuronal HeLa cells.

RepA-WH1 mutant variants were fused to the monomeric fluorescent protein mCherry in a constitutive expression plasmid (see Fig. S1A in the supplemental material). These fusions, referred to here as WH1(WT/A31V/ΔN37)-mCherry for simplification, were the same as those that had been previously validated regarding their aggregation potential and toxicity in E. coli (45–48). While WH1(WT)-mCherry is soluble in the bacterial cytosol and noncytotoxic, the hyperamyloidogenic (A31V)-mCherry variant aggregates and is highly cytotoxic. WH1(ΔN37) is a deletion mutant lacking the amyloidogenic peptide stretch in RepA-WH1 that forms inclusion bodies. When this mutant is expressed in bacteria, it exhibits reduced toxicity compared to WH1(A31V)-mCherry. Cell lines were transfected with the plasmids coding for RepA-WH1 derivatives or mCherry as a control. Soluble fractions of cell lysates were analyzed by Western blotting, 48 h after transient transfection, revealing differing levels of protein expression in the three cell lines tested. The highest expression levels were observed in the N2a cells (Fig. S1B). Variant WH1(ΔN37)-mCherry was not observed in any cell lysate. The N2a cell line was thus selected as an appropriate cell model for further exploring RepA-WH1 prion-like behavior in mammalian cells. As previous work in bacteria had shown that WH1(ΔN37)-mCherry forms massive inclusion bodies (46, 47), we explored the presence of this variant in the insoluble lysate fraction of the N2a cells. WH1(ΔN37)-mCherry was clearly located in the pellet, according to Western blotting (Fig. S1B, right panel), thereby confirming that this mutant also forms insoluble aggregates in the mammalian cytosol.

10.1128/mBio.02937-19.1 FIG S1 Transient expression in mammalian cell lines of distinct variants of the bacterial prion-like protein RepA-WH1 fused to the red fluorescent protein mCherry. (A) Schematic linear representation of the pcDNA3.1 plasmid constructs used to transiently express either the distinct WH1-mCherry fusions or the mCherry control in mammalian cell lines. (B) Assessing the expression of the WH1-mCherry-derived constructions (WT/A31V/ΔN37; ∼55 kDa) or the mCherry control (35 kDa) reported in panel A by Western blotting of soluble lysates 48 h after transient transfection of N2a, SH-SY5Y, and HeLa cell lines. Proteins were detected using an anti-mCherry antibody. Actin (42 kDa) used as a loading control was visualized with an anti-actin antibody. The insoluble fraction (pellet) of cells expressing WH1(ΔN37) in the N2a cells is also shown, compatible with its tendency to form large foci in bacteria (46, 47). Download FIG S1, TIF file, 0.3 MB. Copyright © 2020 Revilla-García et al. 2020 Revilla-García et al. This content is distributed under the terms of the Creative Commons Attribution 4.0 International license.

**FIG 1:**
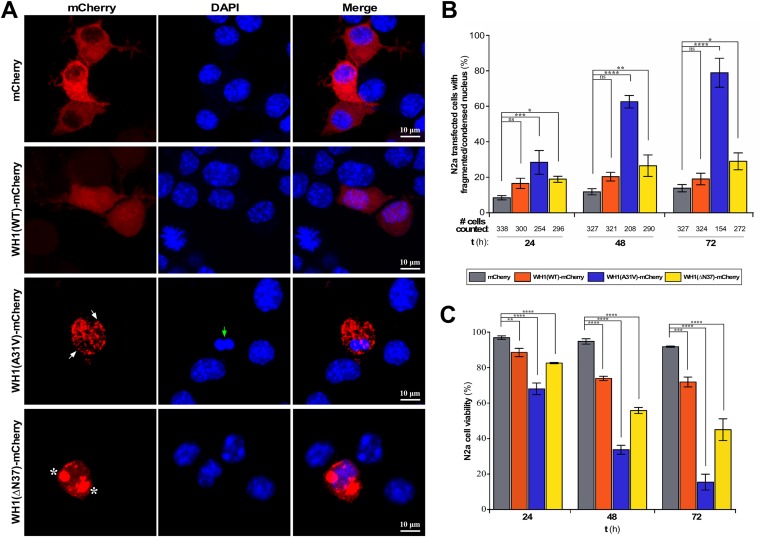
Expression of WH1(A31V)-mCherry in the cytosol of murine N2a cells. (A) Confocal maximum intensity projection images of N2a cells expressing WH1(WT/A31V/ΔN37)-mCherry, or mCherry as a control, 48 h after being transiently transfected (red). White arrows mark the small WH1(A31V)-mCherry fluorescent foci, whereas the large WH1(ΔN37)-mCherry inclusion bodies are indicated by asterisks. Nuclei were stained with DAPI (blue). A green arrow points to an altered nucleus. (B) Quantitative analysis of condensed/fragmented nuclei in N2a transfected cells that expressed for 24, 48, and 72 h either WH1(WT/A31V/ΔN37)-mCherry or the mCherry control. The total number of cells of each type with mCherry fluorescence is displayed below the *x* axis. (C) N2a cell viability, quantitatively estimated from a MTT reduction assay, in response to transient expression of the RepA-WH1 variants for 24, 48, and 72 h. The inferred levels of cytotoxicity of the proteins expressed were as follows: WH1(A31V) ≫ WH1(ΔN37) > WH1(WT) > mCherry. In panels B and C, bars correspond to the mean values from three independent transfections (*n* = 3). One-way ANOVA was performed (*, *P* < 0.1; **, *P* < 0.01; ***, *P* < 0.001; ****, *P* < 0.0001; ns, not statistically significant).

To evaluate the possible cytotoxicity of RepA-WH1, the N2a transfected cells were observed by confocal microscopy. As shown in Fig. 1A, WH1(WT)-mCherry and the mCherry control remained phenotypically soluble (i.e., they showed diffuse cytoplasmic fluorescence) when overexpressed, which demonstrated that none of them has a tendency toward spontaneous aggregation. In contrast, for the WH1(A31V)-mCherry mutant, small red foci were clearly detected in the cytosol, which correlated with its natural propensity to aggregate in bacteria (45–47). Finally, larger intracellular foci, distinct from those seen with WH1(A31V)-mCherry, were observed when WH1(ΔN37)-mCherry was expressed.

Remarkably, inspection of the morphology of nuclei by confocal microscopy revealed the noticeable presence of N2a cells exhibiting a fragmented or condensed nucleus upon WH1(A31V)-mCherry expression, a sign of cell death. This phenotype was rare in cells expressing WH1(WT/ΔN37)-mCherry or the mCherry control (Fig. 1A). Quantitative analysis of the cells showing nuclear condensation/fragmentation at 24, 48, and 72 h after transfection revealed that such differences were statistically significant (Fig. 1B). A substantial decrease in the number of WH1(A31V)-mCherry-expressing cells was detected at 48 h and, in particular, at 72 h after transfection, while the numbers of mCherry control and WH1(WT/ΔN37)-mCherry transfected N2a cells remained roughly the same over time. Such reverse correlations between fragmented/condensed nuclei and cell count support the idea of the cytotoxicity of WH1(A31V).

To further assess the cytotoxic effect of WH1(A31V)-mCherry overexpression on N2a cells, cell viability was estimated by monitoring the activity of NAD(P)H-dependent oxidoreductases 24, 48, and 72 h after transfection (Fig. 1C). A NAD(P)H-dependent tetrazolium salt [3-(4,5-dimethyl-2-thiazolyl)-2,5-diphenyl-2H-tetrazolium bromide {MTT}] reduction assay revealed a decrease in cell viability, compared to the level seen with the mCherry control, for cells expressing either WH1(A31V)-mCherry or WH1(ΔN37)-mCherry. This toxicity was time dependent and was most pronounced for cells expressing WH1(A31V)-mCherry, resulting in a decrease in cell viability of about 60% at 48 h to reach 90% at 72 h. In contrast, no time-dependent changes in cell viability were detected for mCherry and WH1(WT)-mCherry.

**FIG 2:**
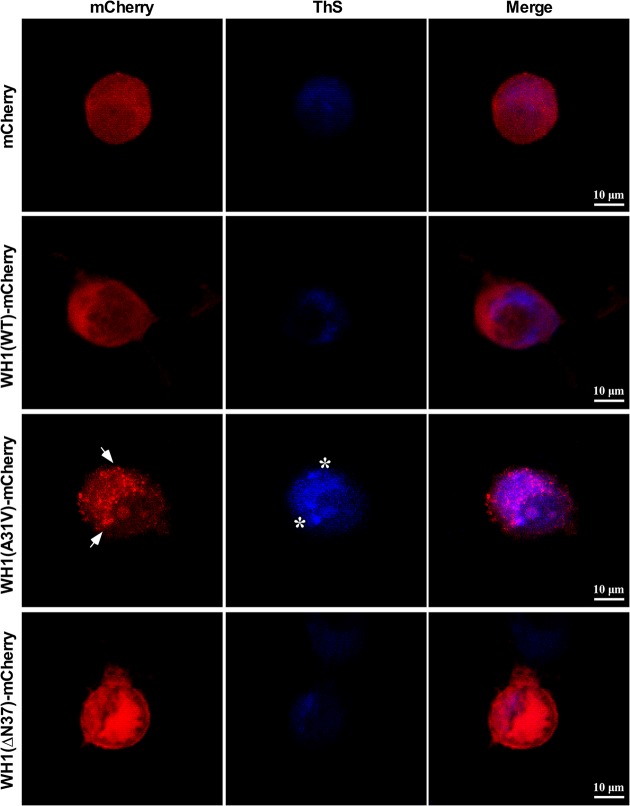
The particles assembled by WH1(A31V)-mCherry in the cytosol of murine N2a cells are amyloids. Confocal maximum intensity projection images of N2a cells transiently expressing for 48 h WH1(WT/A31V/ΔN37)-mCherry or mCherry as the control (red). Cells were stained with ThS (blue) for amyloid detection. Arrows mark examples of the intracellular WH1(A31V)-mCherry puncta, whereas some of the ThS-positive foci are indicated with asterisks. Just the WH1(A31V)-mCherry particles are amyloid by nature.

To verify that the cytotoxic aggregates formed by RepA-WH1 in the N2a cells were amyloid in nature, we employed thioflavin-S (ThS), an amyloidotropic fluorophore that stains RepA-WH1 amyloids in bacteria (45, 46, 54). Forty-eight hours after WH1(WT/A31V/ΔN37)-mCherry or mCherry transfection, staining with ThS was performed and cells were visualized by confocal microscopy (Fig. 2). Image analysis revealed that in the N2a cells expressing WH1(A31V)-mCherry, the aggregated foci were strongly stained by ThS, an indication of their amyloid nature. In contrast, weak (near-background) ThS staining was observed for cells expressing either the mCherry control or the soluble WH1(WT)-mCherry protein and the large WH1(ΔN37)-mCherry aggregates. Reduced binding of the fluorophore was previously considered indicative of the amorphous aggregation of WH1(ΔN37)-mCherry as bacterial inclusion bodies (46).

### *In vitro*-assembled RepA-WH1(A31V) amyloid fibers induce the aggregation of RepA-WH1(WT) transiently expressed in the cytosol of murine cells.

One of the most remarkable properties of amyloid aggregates is their inherent self-propagation potential, being able to template the amyloid conformation on soluble molecules of the same protein. For RepA-WH1, this was shown previously in E. coli *in vivo* by coexpressing WH1(A31V) and WH1(WT) labeled with different fluorescent proteins (47). Thereby, validation of RepA-WH1 as a “generic” model for amyloidosis required testing its ability to seed its self-aggregation within mammalian cells.

**FIG 3:**
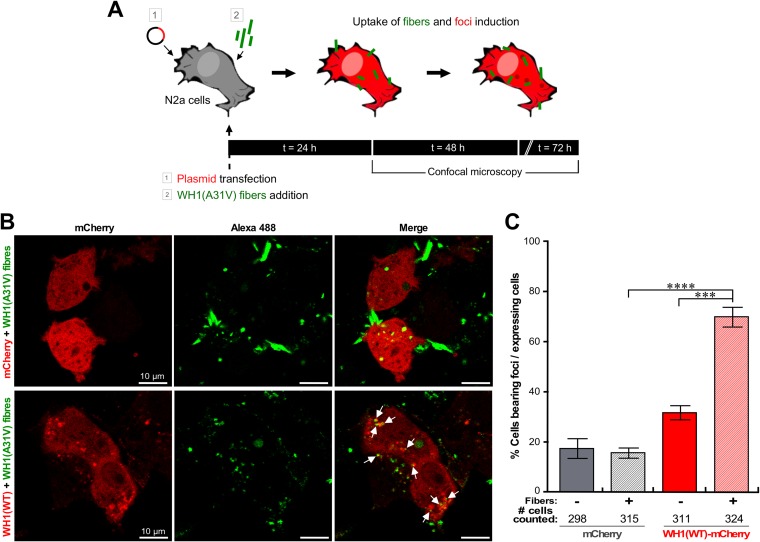
Uptake of *in vitro*-assembled, Alexa 488-labeled WH1(A31V) amyloid fibers by murine N2a cells results in the formation of foci of endogenous WH1(WT)-mCherry. (A) Experimental setup to study the induction of endogenous WH1(WT)-mCherry foci upon uptake of exogenous WH1(A31V) fibers. N2a cells were transiently transfected with plasmids expressing WH1(WT)-mCherry or the mCherry control and immediately incubated with *in vitro*-assembled WH1(A31V) fibers for 24, 48, and 72 h. (B) Confocal section images from the experiment performed as outlined in panel A (48 h after fiber addition, unfixed cells). Exogenous WH1(A31V) fibers (green) were taken up by N2a cells expressing (red) either mCherry (top) or WH1(WT)-mCherry (bottom). Internalized fiber particles which nucleated intracellular WH1(WT)-mCherry amyloid foci are indicated by arrows. (C) Percentage of mCherry-expressing or WH1(WT)-mCherry-expressing N2a cells bearing fluorescent foci 48 h after incubation with WH1(A31V) fibers. The bars in the graph display means of results from three independent experiments (*n* = 3). The total number of cells of each type counted is displayed below the *x* axis. For statistical analysis, Student’s *t* test was performed (***, *P* < 0.001; ****, *P* < 0.0001).

We explored whether exogenous, *in vitro*-assembled WH1(A31V) fibers (Fig. S2), whose amyloid nature has been well established (40, 43, 45, 55, 56), could trigger the aggregation of the otherwise soluble WH1(WT) protein (Fig. 1A, second row) in the cytosol of mammalian cells. To this end, N2a cells transiently expressing WH1(WT)-mCherry in the cytosol were exposed to *in vitro*-preassembled WHI(A31V)-Alexa 488 labeled amyloid fibers [for simplification, referred to here as WH1(A31V) fibers] (Fig. 3A). These were mechanically fragmented before incubation with cells to get fiber sizes more suitable for their uptake (see Materials and Methods) (Fig. S2). To check for the specific requirement of the WH1(WT) domain as the target for aggregation templating by WH1(A31V) fibers, control cells expressing just mCherry were incubated with the fibers in parallel. Confocal microscopy sections revealed that the WH1(A31V) fiber particles were efficiently internalized by the N2a cells expressing either mCherry or WH1(WT)-mCherry (Fig. 3B). In the latter, fibers colocalized with the endogenous WH1(WT)-mCherry in small, dot-like cytosolic aggregates (Fig. 3B, arrows). Approximately 70% of the WH1(WT)-mCherry-expressing cells displayed red puncta upon incubation with the fibers, while spontaneous focus formation of WH1(WT)-mCherry was detected in <35% of the cells after 48 h (Fig. 3C). These figures significantly differed from the <20% of focus-bearing cells counted for the mCherry control, irrespective of the presence or absence of added fibers (Fig. 3C). Overall, these observations support the idea that induction of foci by WH1(A31V) amyloid fibers requires expression of a homologous protein substrate in the recipient cells.

10.1128/mBio.02937-19.2 FIG S2 *In vitro*-assembled and Alexa 488-labeled WH1(A31V) fibers used for cocultivation with the murine N2a cells. (A) Negative-staining transmission electron microscopy (TEM) micrographs of the fibers before (left) and after (right) being disrupted by sonication, previous to their addition to the transfected N2a cells. (B) Confocal section of the fiber samples. Green fluorescence from the tracer Alexa 488-labeled WH1(A31V) molecules incorporated into the fibers is evident. Download FIG S2, TIF file, 0.9 MB. Copyright © 2020 Revilla-García et al. 2020 Revilla-García et al. This content is distributed under the terms of the Creative Commons Attribution 4.0 International license.

**FIG 4:**
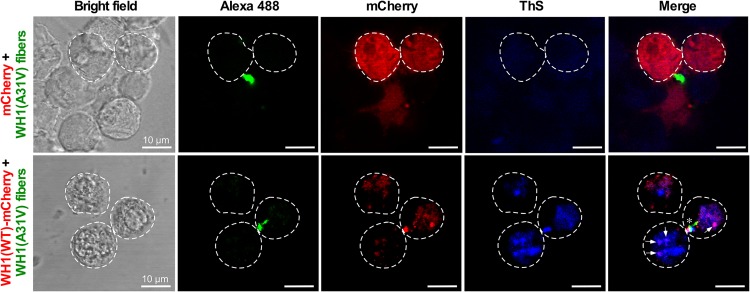
Uptake of WH1(A31V) fibers induced WH1(WT)-mCherry amyloid formation in the cytosol of murine N2a cells. Confocal sections of N2a cells expressing soluble mCherry or WH1(WT)-mCherry (red), incubated for 48 h with *in vitro*-assembled, Alexa 488-labeled WH1(A31V) fibers (green) are shown. Cells were then stained with ThS (blue) for amyloid detection. Bright-field projection views of the cells under study are displayed on the left. Transfected cells that were placed within each confocal section are encircled with dashed lines. In the merged images, coincident red and blue puncta are indicated by arrows, while the asterisk indicates the colocalization of a fiber and an intracellular aggregate stained by ThS.

As a consequence of amyloid templating, it can be assumed that the foci induced in mammalian cells by the internalized amyloid fibers are amyloid aggregates. In order to qualitatively assess this point, ThS staining was performed (Fig. 4). Confocal microscopy images obtained after 48 h of fiber addition to the cell cultures revealed selective binding of ThS to the induced cytosolic WH1(WT)-mCherry aggregates. In contrast, weak ThS staining was observed for cells expressing mCherry, most likely coming from background fluorescence. These findings are compatible with the results of ThS binding to the intracellular aggregates generated upon transient expression of WH1(A31V)-mCherry (Fig. 2). Therefore, ThS staining provides evidence for the amyloid nature of the WH1(WT)-mCherry aggregates as templated by the WH1(A31V) fibers in the cytoplasm of murine N2a cells.

### RepA-WH1(A31V) released from murine cells can cross-seed amyloid aggregation in the cytosol of recipient human cells stably expressing RepA-WH1(WT).

The studies described above provided evidence for the ability of WH1(A31V) to cross-seed WH1(WT) amyloidogenesis in the cytosol of mammalian cells. To further explore this, we generated stable donor cell lines in which the WH1 variants and controls, fused to the green fluorescent protein (GFP) TagGFP2, are expressed under the control of an inducible promoter (pTRE3G Tet-ON vector, genome-integrated THN–transactivator protein [rtTA]; see Materials and Methods) (Fig. S3A). We chose SH-SY5Y as the parental donor cell line as transgene expression in that background was less pronounced than in N2a cells (Fig. S1B). Unfortunately, we were unable to isolate any clone expressing WH1(A31V)-TagGFP2, suggesting that, even under conditions of tightly regulated gene expression, any transcriptional leak of this mutant variant results in acute cytotoxicity. For the WH1(WT)-TagGFP2 and the control TagGFP2 constructs, efficient doxycycline (Dox)-induced expression was verified through Western blotting (Fig. S3B) and flow cytometry (Fig. S3C). Confocal microscopy imaging indicated that both the THN-rtTA/WH1(WT)-TagGFP2 and THN-rtTA/TagGFP2 doubly stable clones showed homogeneous, diffuse expression throughout the cytosol (Fig. S3D).

10.1128/mBio.02937-19.3 FIG S3 Experimental setting used for the stable expression of proteins in the human neuroblastoma cell line SH-SY5Y. (A) Tet-ON, doxycline (Dox)-dependent expression cascade from the integrated pCMV-Tet3G transactivator (rtTA; left) and the pTRE3G response plasmids (right). (B) Western blots of protein extracts from THN-rtTA/SH-SY5Y cells expressing WH1(WT)-TagGFP2 or TagGFP2 upon induction for 48 h with 0.5 μg/ml Dox. Proteins were probed with an anti-GFP antibody, whereas detection of vinculin (∼123 kDa) was used as a loading control. (C) Flow cytometry histograms (FL1 channel) highlighting the population of live THN-rtTA/SH-SY5Y cells expressing (green) or not expressing (gray) the engineered fluorescent proteins after 48 h of culture in the presence of Dox (see panel B). Mean fluorescence intensity (MFI) of TagGFP2, 3.44; MFI of WH1(WT)-TagGFP2, 1.61. (D) Confocal maximum intensity projection images of THN-rtTA/SH-SY5Y clones stably expressing the rtTA transactivator and TagGFP2, or WH1(WT)-TagGFP2 (green), 48 h after Dox addition. Nuclei were visualized using DAPI (blue). Both proteins are expressed in soluble form (diffuse fluorescence) in the cytoplasm. Download FIG S3, TIF file, 1.3 MB. Copyright © 2020 Revilla-García et al. 2020 Revilla-García et al. This content is distributed under the terms of the Creative Commons Attribution 4.0 International license.

**FIG 5:**
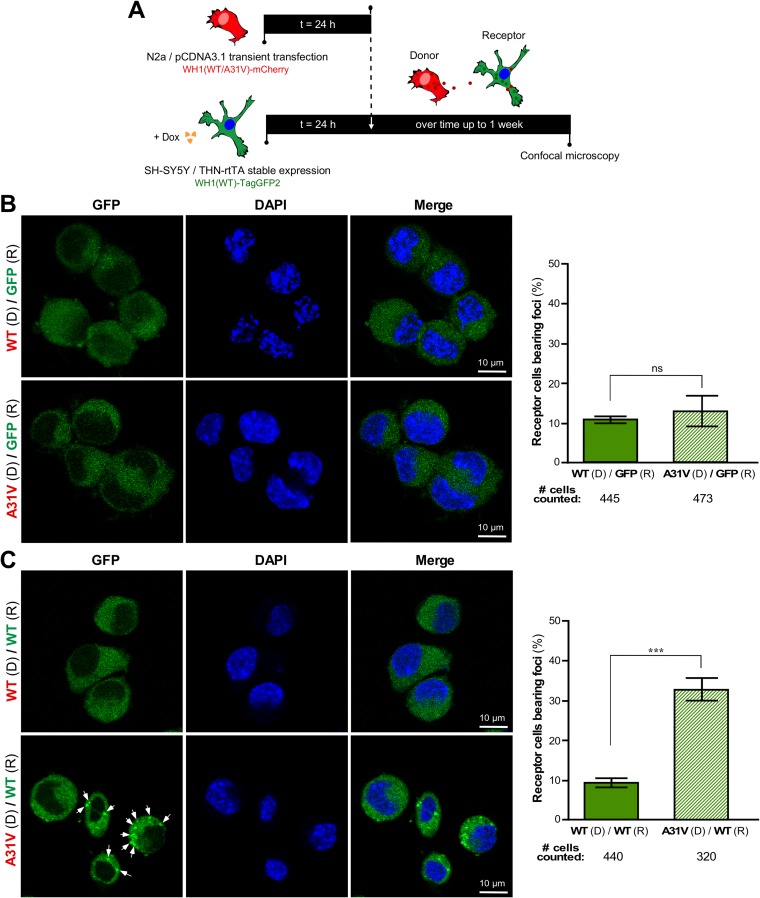
Uptake by human SH-SY5Y cells of WH1(A31V)-mCherry generated by murine N2a cells results in formation of foci of endogenously expressed WH1(WT)-TagGFP2. (A) Experimental setup to study horizontal propagation of WH1(A31V) between cell lines. At 24 h after being transfected with plasmids, donor N2a cells transiently expressing soluble WH1(WT)-mCherry, or the amyloidogenic WH1(A31V)-mCherry, were cocultured for up to 7 days with THN-rtTA/SH-SY5Y receptor cells stably expressing, upon Dox induction, either WH1(WT)-TagGFP2 or TagGFP2. (B) (Left) Confocal sections of cells in cultures assayed as described for panel A in which the receptor cells, R, were expressing the TagGFP2 control, while the donor cells, D, were expressing either WH1(WT)-mCherry or WH1(A31V)-mCherry. No large fluorescent puncta were observed after 5 days. DAPI staining of nuclear DNA was carried out. (Right) Percentage of THN-rtTA/SH-SY5Y/TagGFP2 receptor cells, R, bearing intracellular foci after 5 days of coculture with transiently transfected WH1(WT)-mCherry or WH1(A31V)-mCherry N2a donors, D. (C) (Left) Confocal sections of cells in cultures assayed as described for panel B, but in which the receptors, R, were expressing WH1(WT)-TagGFP2 instead of just TagGFP2. Bright green spots (arrows) were differentially promoted by the cells donating WH1(A31V)-mCherry, but not by those expressing WH1(WT)-mCherry. (Right) Quantitative analysis of intercellular propagation of RepA-WH1. Percentage of THN-rtTA/SH-SY5Y/WH1(WT)-TagGFP2 receptor cells, R, bearing intracellular foci after 5 days of coculture with transiently transfected WH1(WT)-mCherry or WH1(A31V)-mCherry N2a donors, D. Bars displayed in both histograms represent means of results from three independent experiments (*n* = 3). The total number of cells of each type counted is displayed below the *x* axes. For statistical analysis, Student’s *t* test was performed (***, *P* < 0.001; ns, not statistically significant).

The horizontal, cell-to-cell spreading of RepA-WH1, a key feature qualifying it as a prion-like protein (21), was then explored. To this end, murine N2a donor cells transiently expressing either WH1(A31V)-mCherry or WH1(WT)-mCherry (Fig. 1A) were cocultured with THN-rtTA/WH1(WT)-TagGFP2 recipient cells, which stably expressed soluble WH1(WT)-TagGFP2 upon Dox addition (Fig. S3D), for a period of up to a week (Fig. 5A). To control for nonspecific spontaneous aggregation in the human cells, recipient cultures not mixed with donors were similarly processed in parallel. The formation of foci in the recipient cells was assessed by confocal microscopy imaging every 24 h (Fig. 5B and C, left). Noticeably, small green fluorescent puncta were detected over time in the THN-rtTA/WH1(WT)-TagGFP2 recipient cells when the donor N2a cells expressing the hyperamyloidogenic variant WH1(A31V)-mCherry were added (Fig. 5C, left, second row), peaking at 5 days and decreasing thereafter, which suggests killing of the cells. In contrast, no substantial formation of WH1(WT)-TagGFP2 foci was observed upon addition of the N2a donors transiently expressing WH1(WT)-mCherry (Fig. 5C, left, first row) to the human recipient cells. Fluorescent puncta were also not apparent for their control counterparts, in which donors expressing WH1(A31V/WT)-mCherry were added to cultures of recipient cells expressing THN-rtTA/TagGFP2 (Fig. 5B, left). These results fully parallel (and strengthen) the observations made when exogenously added, *in vitro*-assembled WH1(A31V) fibers induced the formation of WH1(WT)-mCherry foci in the N2a cells (see above and Fig. 3). Five-day cocultures were selected for quantitative image analysis of the fraction of cells that exhibited green fluorescent foci. Just 10% to 12% of the human control cells endogenously expressing TagGFP2 showed some fluorescent spots, irrespective of whether they had been cocultured with murine cells expressing WH1(A31V)-mCherry or with those expressing WH1(WT)-mCherry. Puncta were apparent in ∼35% of the recipient cells expressing WH1(WT)-TagGFP2 only if the hyperamyloidogenic WH1(A31V)-mCherry variant was expressed by the donor murine cells (Fig. 5B and C, right).

While WH1(A31V)-mCherry aggregates must first be released (either through exocytosis or, more likely, upon cell death) to the culture medium from the donor cells and subsequently gain access into the recipient cells, it is noteworthy that red fluorescent WH1(A31V)-mCherry particles were rarely detected at the intercellular space. Although it is possible that loss of such particles may occur during the manipulation of the samples (e.g., for cell passages or microscopy), a likely explanation is that their size range is beyond the resolution of conventional confocal imaging (25, 26).

In conclusion, these results showed significant differences in the intercellular infectivity of soluble WH1(WT)-mCherry and hyperamyloidogenic WH1(A31V)-mCherry. The cells suffering from RepA-WH1(A31V) amyloidosis were highly proficient in the propagation of an aggregation-related phenotype, which was consistent with the results obtained for the *in vitro*-preassembled RepA-WH1(A31V) fibers. Taken together, these parallel observations suggest that the bacterial RepA-WH1 protein indeed exhibits a prion-like mechanism for horizontal transmission in mammalian cells.

### A proteomic outline of the cytotoxicity of RepA-WH1(A31V) amyloids in mammalian cells.

In a previous study, we characterized the system-wide response of E. coli to WH1(A31V)-mCherry expression to identify pathways potentially affected by the bacterial amyloidosis (48). To assess cellular responses to the toxic WH1(A31V) amyloids, we performed parallel proteomic studies on the following three groups of samples: (i) N2a cells transiently expressing WH1(WT)-mCherry or mCherry, collected after 48 h of incubation with WH1(A31V) fibers (Fig. 3; see also Data Set S1); (ii) THN-rtTA/SH-SY5Y cells expressing WH1(WT)-TagGFP2 or TagGFP2, harvested after 5 days of induction with Dox in coculture with N2a cells expressing WH1(A31V)-mCherry (Fig. 5; see also Data Set S2); and (iii) control naive N2a or THN-rtTA/SH-SY5Y cells.

10.1128/mBio.02937-19.5 DATA SET S1 Electrospray ionization-tandem mass spectrometry (ESI-MS/MS) analysis of the proteome response of mouse N2a cells to WH1(A31V) fiber uptake-promoted amyloidogenesis. Download Data Set S1, XLSX file, 2.9 MB. Copyright © 2020 Revilla-García et al. 2020 Revilla-García et al. This content is distributed under the terms of the Creative Commons Attribution 4.0 International license.

10.1128/mBio.02937-19.6 DATA SET S2 ESI-MS/MS analysis of the proteome response of human THN-rtTA/SH-SY5Y cells seen on transmission of WH1(A31V)-mCherry from murine N2a cells. Download Data Set S2, XLSX file, 2.8 MB. Copyright © 2020 Revilla-García et al. 2020 Revilla-García et al. This content is distributed under the terms of the Creative Commons Attribution 4.0 International license.

**FIG 6:**
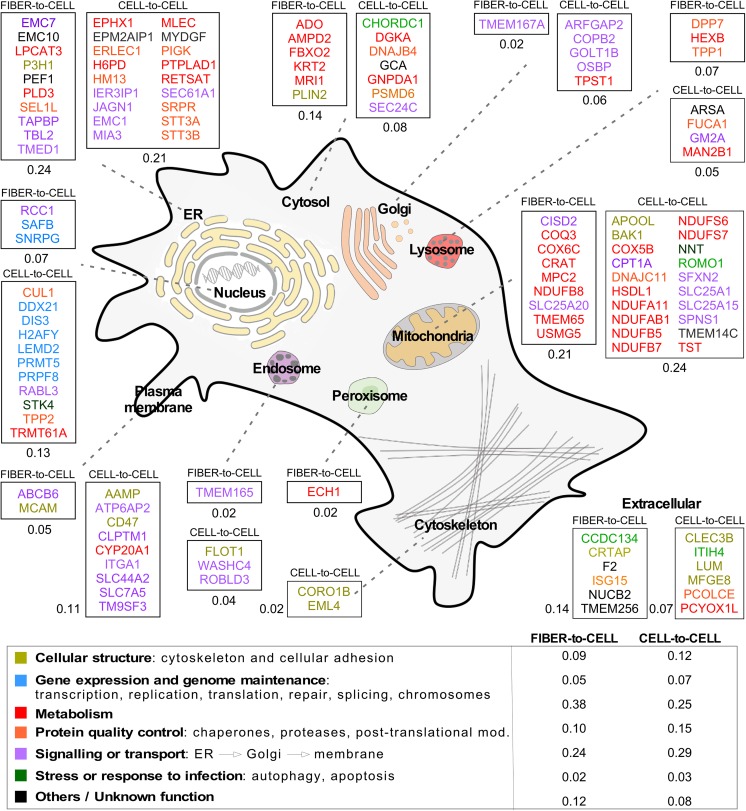
Proteome-wide changes in mammalian cells expressing WH1(WT) and exposed to either *in vitro*-assembled WH1(A31V) fibers or WH1(A31V)-mCherry released from cocultured cells. The cartoon displays, overlaid on the sketch of a mammalian cell, the names of the proteins differentially and uniquely found in cells expressing WH1(WT)-mCherry, following exposure to fibers or cell-derived WH1(A31V). Proteins are grouped in boxes according to their subcellular location, as indicated by gene ontology. Numbers beside each box indicate the fraction of proteins predicted to reside in that specific cellular compartment in relation to the total number of unique proteins identified under the corresponding experimental conditions. The color coding refers to the main function reported in the literature for these proteins (see the legend at the bottom, including the fraction of them placed in each functional group). ER, endoplasmic reticulum.

The subsets of the proteomes that were exclusively found in recipient cells expressing WH1(WT)-mCherry (murine N2a cells) or WH1(WT)-TagGFP2 (human THN-rtTA/SH-SY5Y cells), and not in cells expressing only their respective fluorescent tag controls, upon fiber-induced or cell-to-cell spread of amyloidogenesis reflect a broader response for the latter experimental scenario (Fig. 6; see also Fig. S4). Albeit the individual proteins identified are not the same (perhaps due to host species-specific features), both data sets point to shared pathways affected in the RepA-WH1 amyloidosis. These include distinct subcellular organelles (in particular, mitochondria and the endoplasmic reticulum) and processes, among which metabolism and signaling are the most frequent hits. Cluster analyses further refine these findings, confirming that mitochondria are at the very core of the RepA-WH1 amyloidosis in both experimental approaches (Fig. S4), with complex I of the respiratory chain (NADH:ubiquinone oxidoreductase) identified as the most prominent node. In addition, protein quality control machinery (the proteasome, ubiquitin-ligases, and chaperones) and cell signaling and intracellular trafficking networks linked to protein secretion are also affected by WH1(A31V) uptake.

10.1128/mBio.02937-19.4 FIG S4 Systems analysis of the response of murine N2a (A) and human THN-rtTA/SH-SY5Y (B) cells, expressing soluble WH1(WT) or its fluorescent tags, to cocultivation with the *in vitro*-assembled and the cell-released WH1(A31V) amyloids, respectively. Venn diagrams (top) indicate the number of proteins exclusively identified in the WH1(WT)-mCherry/TagGFP2 cells or in the control mCherry/TagGFP2 cells (Fig. 6), as well as in both, over those found in the naïve N2a or THN-rtTA/SH-SY5Y backgrounds, which were subtracted. STRING v.11 (74) functional clustering analysis (bottom; inset, program settings) was performed, using all available prediction sources, for the proteins identified in either experimental set. After removal of the unconnected nodes, the presence of the remaining clusters, similar in the two amyloid transmission scenarios, points to contributions of the mitochondrial membrane respiratory complexes, protein quality control, and signaling/trafficking to the RepA-WH1 “generic” model of amyloidosis in mammalian cells. Download FIG S4, TIF file, 2.7 MB. Copyright © 2020 Revilla-García et al. 2020 Revilla-García et al. This content is distributed under the terms of the Creative Commons Attribution 4.0 International license.

Interestingly, similarities between the responses of mammalian mitochondria to amyloid-promoted neurodegeneration and how E. coli reacts to RepA-WH1 amyloidosis were previously proposed on the basis of systems analyses carried out in the latter host (48). The results presented here reinforce this view and make sense in light of the common ancestry of Gram-negative bacteria and mitochondria, validating RepA-WH1 as a minimal, autonomous proxy that can be used to sketch core mitochondrial routes involved in human amyloidosis.

## DISCUSSION

The ability of protein aggregates to intercellularly template their amyloid structure on soluble molecules of the same protein found in naive recipient cells is a hallmark of human neurodegenerative diseases (16–20). The intercellular traffic of these prion-like proteins or prionoids is likely mediated by generation of extracellular vesicles from the donor cells and subsequent endocytic uptake by the recipient cells (26).

RepA-WH1 is a bacterial protein that recapitulates in E. coli many of the essential features of an amyloid proteinopathy, including the formation of cytotoxic amyloid aggregates (45–48), the existence of strains that give rise to distinct phenotypes, and their epigenetic vertical (mother-to-daughter) transmission (46). However, its ability to be horizontally (cell to cell) transmitted as a prion-like protein had not been addressed yet in bacteria, due to the stringent barrier to the uptake of large protein aggregates imposed by the peptidoglycan and, in Gram-negative bacteria, also by the outer membrane. We have now used mammalian cultured cells and fluorescently labeled proteins to address the issue of the horizontal transmissibility of RepA-WH1. We first transiently expressed distinct variants of RepA-WH1 in murine cells, which led to the formation of amyloids with degrees of cytotoxicity similar to those described in bacteria, i.e., WH1(A31V) ≫ WH1(ΔN37) > WH1(WT) (46, 48). Then, incubation of those cells with *in vitro*-assembled amyloid WH1(A31V) fibers showed their ability to induce the formation of foci by WH1(WT), which is otherwise soluble as when expressed in the cytoplasm of the recipient cells. In a subsequent step, we generated a stable and inducible cell line expressing soluble WH1(WT), which assembled as fluorescent puncta upon exposure to WH1(A31V) aggregates generated by donor cells. The results from these experiments fulfill the last stalling requirement to qualify RepA-WH1 as a prion-like protein: its ability to be horizontally transmissible.

It is noteworthy that, under our experimental conditions, the uptake of recombinant WH1(A31V) amyloids by cells not expressing WH1(WT), but by cells expressing just the fluorescent proteins to which the prion-like protein was tagged, did not result in any evident toxicity for the recipient cells. There is no protein with a noticeable degree of sequence identity with RepA-WH1 in the human proteome (BLAST best match E value = 0.11). However, it has been recently suggested that bacterial extracellular amyloids from gut microbiota may template amyloidogenesis on human proteins implicated in neurodegeneration in spite of the lack of significant sequence similarities (32–35). This could constitute a matter of concern with respect to the biosafety of bacterial amyloids. In contrast, the inability of WH1(A31V) amyloids to induce either intracellular aggregation or toxicity in naive mammalian cells not expressing RepA-WH1 qualifies this protein as biosafe.

In the context of the experiment in which amyloidogenesis was induced by adding exogenous fibers, it is most relevant that the intracellular foci in the recipient cells were templated by a well-characterized amyloid structure assembled *in vitro* (40, 43), which in turn was seeded by intracellular aggregates extracted from E. coli cells experiencing WH1(A31V) amyloid toxicity (45). Therefore, it can be assumed (6, 13, 57–60) that the intracellular amyloid (ThS-reactive) foci in the recipient murine cells might have the same intimate structure as the aggregates reported to kill bacterial cells.

The toxicity of the amyloid proteins involved in neurodegeneration can be due to the loss, upon their aggregation, of an essential cellular function and/or to the gain of an emergent functionality by the aggregates (2), e.g., upon the assembly of oligomeric membrane pores (42). In the case of the phenotype caused in the mammalian cells by the expression of WH1(A31V) amyloids (Fig. 1 and 2), or by their uptake and subsequent templating on WH1(WT) in the recipient cells (Fig. 3 and 5), cytotoxicity is necessarily an intrinsic property of the aggregates themselves, because, *a priori*, no function is associated with this bacterial prion-like protein in the heterologous mammalian host. Very recently, RepA-like proteins with a conserved WH1 domain (60) were described as nearly the only gene products encoded by small mobile genetic elements that were isolated in dairies and in meat (bovine meat and milk factors [BMMFs]), with a probable origin in cattle microbiota, but also in samples from patients with cancer and multiple sclerosis (61). These findings open the intriguing possibility that the cytotoxicity described here for RepA-WH1 might have broader relevance in human disease.

Mass spectrometry (MS) analyses of the proteins specifically detected upon the uptake of RepA-WH1(A31V) in the two main scenarios of experimental transmission addressed (Fig. 6) provided a picture sharing some similarities to cellular events in neurodegeneration. In the case of exogenous nucleation of WH1(WT) amyloids by WH1(A31V) fibers, proteins annotated as involved in neurodegenerative diseases were identified (Crat, Hexb, Pld3, and Sel1l), while other proteins were annotated as related to ubiquitin ligases and proteasome-mediated degradation pathways (Fbxo2 and Isg15). Upon the uptake of secreted/released WH1(A31V) particles, proteins linked to neurodegeneration (Arsa, Epm2aip1, and Hm13) were identified too, together with cochaperones (Chordc1, Dnajb4, and Dnajc11) and proteins involved in vesicular trafficking (Arfgap2, Copb2, Dis3, and Flot1) or in apoptosis (Bak, Cul1, Ier3ip1, Mfge8, Psmd6, Tpp2, and Stk4). It is noteworthy that Stk4 participates in chromatin condensation, a phenotype observed upon WH1(A31V) expression (Fig. 1B). Mitochondrial proteins and proteins located at the endoplasmic reticulum were the most common hits, preferentially related to bioenergetics (respiratory chain) and to secretory pathways, respectively. Two proteins are worth noting in the context of what is known on RepA-WH1(A31V) toxicity in E. coli: Apool, which binds to cardiolipin, the bacterial and mitochondrion-specific acidic phospholipid that promotes the formation of pores by the prionoid in model membranes (42), and Romo1, a protein that enhances the formation of reactive oxygen species (ROS) in mitochondria, usually as an antimicrobial response. Romo1 might be playing the same role, albeit through a different mechanism, as that played by the auto-oxidation of the alternative dehydrogenase NdhII in bacteria, i.e., constituting the first step in the oxidative cascade leading to cell death (48). Attempts to counteract ROS generation in mitochondria seem to rely on Nnt, which generates NADPH and thus the power of reduction to detoxify free radicals. Interestingly, a central role in the cytotoxicity of the PrP prion has been proposed for NADPH oxidases (62).

Several systems analyses, based on differential transcriptomics and proteomics, have been reported for conditions related to neurodegenerative amyloidosis (52, 63–69). One-third of the proteins identified in our analysis (Fig. 6) were also found among the genes overexpressed, or among the proteins captured within aggregates, in those data sets, thus defining a subset of proteins (see Table S1 in the supplemental material) representative of a generic cellular model of amyloidosis, as it can be outlined with the synthetic bacterial prion-like protein RepA-WH1.

10.1128/mBio.02937-19.7 TABLE S1 Subset of proteins in Data Sets S1 and S2 matching proteins/genes found in published system analyses of aggregation-triggered neurodegeneration. Download Table S1, XLSX file, 0.1 MB. Copyright © 2020 Revilla-García et al. 2020 Revilla-García et al. This content is distributed under the terms of the Creative Commons Attribution 4.0 International license.

## MATERIALS AND METHODS

### Construction of plasmids. (i) Generation of the pcDNA3.1 vectors.

The gene fusions *repA-WH1*(*WT*)*-mCherry*, *repA-WH1*(*A31V*)*-mCherry*, and *repA-WH1*(*ΔN37*)*-mCherry* or *mCherry* were amplified by PCR (5′-CCGGGTACCATGGGCAGCAGCCATCATC and 5′-CGGGAATTCTTACTTGTACAGCTCGTCCAT primers) (*Pfu* DNA polymerase; Promega) from pRG*rectac*-NHis constructions (45). The PCR products were cloned into pcDNA3.1 (Invitrogen) after cutting was performed with KpnI (5´ end) and EcoRI (3´ end) (see Fig. S1A in the supplemental material). Constructs were screened by colony PCR and restriction enzyme digestion and were confirmed by Sanger sequencing (Secugen, Spain).

### (ii) Generation of the pTRE3G vectors.

For generation of SH-SY5Y stable cell lines expressing WH1(WT)-TagGFP2, or TagGFP2 as a control, the *TagGFP2* sequence of pTagGFP2-N vector (Evrogen) was amplified via PCR (5′-ATAAGAATTCCGGAATGAGCGGGGGCGAGGAG and 5′-CGGGGAATTCCATATGTTACCTGTACAGCTC primers) and cloned into pcDNA3.1-mCherry (see above) via BspEI and EcoRI digestions, thus replacing the red by the green fluorescent protein. The RepA-WH1(WT) domain was then excised from pcDNA3.1-*WH1*(*WT*)*-mCherry* and fused in frame with TagGFP2 by digestion with SacII and BspEI. For the Tet-ON-inducible expression plasmid pTRE3G (Clontech), *WH1*(*WT*)*-TagGFP2* and *TagGFP2* genes were amplified by PCR (5′-GAAGATCTATGGGCAGCCATCATCATCA and 5′-CCGGAATTCCATATGTTACCTGTACAGCCTC primers). The amplified PCR fragments were subcloned into pTRE3G with BglII and NdeI to obtain pTRE3G-*TagGFP2* and pTRE3G-*WH1*(*WT*)*-TagGFP2* (Fig. S3A).

### (iii) Generation of pET-WH1(A31V,K132C).

For labeling the protein RepA-WH1(A31V) with Alexa Fluor 488, a variant with only one cysteine was constructed. The *repA*-WH1(A31V) gene was amplified from a mutant variant in which Cys29 and Cys106 had been replaced by serines (70), and a new C-terminal cysteine was added replacing Lys in position 132, using the following primer: 5′-GCTAAGCTTCAACAGAGCTGATAGCTGGTGAACTC. The *repA*-*WH1*(*A31V*, *K132C*) gene was interchanged with the insertion in pET3d-*repA-WH1* (49) upon digestion with SacII and BamHI.

### Protein biochemistry. (i) RepA-WH1(A31V) protein purification.

The RepA-WH1(A31V) protein used in the fibrillation studies was purified as described previously (40). RepA-WH1(A31V,K132C) was expressed in E. coli BL21(DE3) bearing pET-*WH1*(*A31V*,*K132C*) plus pLysS to enable endogenous lysozyme-promoted lysis after freezing-thawing. Cells were grown in Terrific Broth medium supplemented with ampicillin (Ap) to 100 μg⋅ml^−1^ at 30°C to an optical density at 600 nm (OD_600_) of 0.8. Then, IPTG (isopropyl-β-d-thiogalactopyranoside) was supplied to reach a concentration of 0.5 mM and expression proceeded for 6 h at room temperature (RT). Cells were harvested and the protocol was followed as described previously (40). The concentration of purified protein was determined by absorption at 280 nm (A = E ⋅ C ⋅ I; C, Molar concentration; I, path length in cm; ε^280^ = 11,548 M^−1^⋅cm^−1^).

### (ii) Fluorescent labeling of RepA-WH1.

The single cysteine residue in RepA-WH1(A31V,K132C) was labeled with Alexa Fluor 488 maleimide (Invitrogen) using thiol chemistry. Briefly, the protein (∼50 μM) was dialyzed against 20 mM sodium phosphate buffer (pH 7.0) and 100 mM Na_2_SO_4_. After dialysis, TCEP [Tris(2-carboxyethyl) phosphine-hydrochloride] was added to reach a concentration of 2 mM and urea to reach a concentration of 2 M and the reaction mixture was incubated for 1 h on ice. TCEP maintains the cysteine residue in a reduced form, available for derivatization. The fluorophore was prepared by dissolving 250 μg in 10 μl H_2_O and was added dropwise to the protein solution in a small vial under conditions of gentle agitation. We used a 5-fold molar excess of the dye to protein at 50 μM. From that point onward, the sample was protected from light using aluminum foil. The conjugation reaction was allowed to proceed at RT for 4 h under conditions of mild agitation. After protein labeling, unconjugated Alexa Fluor 488 was separated by the use of a 5-ml desalting column (GE Healthcare). The sample was divided into aliquots, flash frozen in N_2_(l), and stored at −70°C after addition of glycerol to reach a concentration of 10%. Each aliquot was thawed immediately before the experiment and used only once. The fraction of protein labeled was 20% (i.e., ∼0.2 mol of dye per mole of protein).

### Fiber assembly.

Fibers were prepared with a modification of a previously described protocol (40, 43). RepA-WH1(A31V) (25 μM) and Alexa 488-labeled RepA-WH1(A31V,K132C) (0.5 μM) were mixed in a final reaction volume of 100 μl of 0.1 M Na_2_SO_4_, 4 mM MgSO_4_, 40 mM HEPES (pH 8), and 7% polyethylene glycol (PEG) 4000. Purified RepA-WH1(A31V)-mCherry bacterial inclusions (1 μl [diluted 1:100 from the stock]) were added to nucleate amyloid assembly (45). Fibers were formed in 500 μl Eppendorf tubes at 4°C for 20 days. For cellular uptake experiments, fibers were centrifuged at 13,000 rpm for 30 min. After centrifugation, the supernatant was removed and the fibers were washed with buffer (50 mM Na_2_SO_4_, 40 mM HEPES [pH 8]) and centrifuged again followed by a final resuspension in the same buffer. To ensure a more efficient process of internalization, immediately before incubation with cells, fibers were fragmented by 5 min of sonication in a water bath (USC100T ultrasonic cleaner [60 W]) and were further broken up by pipetting with a 100-μl Hamilton syringe for 2 min. Fibers were checked by means of electron microscopy (Fig. S2A), as indicated previously (40, 42), and confocal microscopy (Fig. S2B; see below). The size distribution of the fibers as added to the cell cultures was 257.6 ± 188.0 nm (*n* = 243).

### Whole-cell protein extraction.

Either transiently transfected N2a or stable SH-SY5Y cells (see below) were rinsed in phosphate-buffered saline (PBS), detached by scraping, and harvested by centrifugation at 2,000 rpm for 5 min at 4°C. Cell pellets were resuspended in 100 μl of radioimmunoprecipitation assay (RIPA) buffer (Sigma), supplemented with protease and phosphatase inhibitors (Roche), incubated on ice for 30 min, and then centrifuged at 14,000 rpm for 5 min at 4°C. Pellets and supernatants were kept frozen at −70°C. Cell lysate supernatants were denatured with Laemmli buffer for 5 min at 95°C, and samples were loaded (30 μg total protein per lane) onto 12.5% SDS-denaturing polyacrylamide gels. Western blotting was performed after semidry transference of SDS-PAGE gels to polyvinylidene difluoride (PVDF) membranes, using mouse anti-GFP (1:1,000; Abcam), or rabbit anti-mCherry (1:5,000; Abcam) antibodies, with anti-actin (1:1,000; Abcam) or anti-vinculin (1:1,000; Sigma) antibodies used as loading controls. The membranes were then incubated with horseradish-peroxidase (HRP)-conjugated secondary anti-mouse/rabbit antibodies (1:10,000; Sigma). Blots were developed with ECL (Pierce).

### Cell biology. (i) Cell cultures.

The murine neuroblastoma Neuro 2a (N2a; DSMZ-Germany) and the human HeLa (DSMZ) and neuroblastoma SH-SY5Y (CIB-CSIC stock) cell lines were cultured in Dulbecco’s modified Eagle medium in a mixture containing nutrient mixture F-12 (DMEM-F12) (Gibco) supplemented with 10% tetracycline-free fetal bovine serum (FBS) (Capricorn), 200 mM glutamine (Gibco), and 1% penicillin/streptomycin (Gibco). For selection and maintenance of SH-SY5Y stable clones, Geneticin (Gibco) and hygromycin B (Clontech) were added to the medium. Tet-ON expression was induced by doxycycline (Sigma). Cultures of cells were carried out at 37°C in a humidified 5% CO_2_ incubator. Every 3 to 4 days, cells were grown to near-confluence (80% to 90% density) and then detached using 0.05% trypsin-EDTA (Gibco) for subculturing.

### (ii) Transfection of N2a cells.

N2a cells were transiently transfected at ∼70% confluence in a 6-well plate by adding (per well) 10 μl Lipofectamine 2000 (Invitrogen) together with 3 μg of the corresponding pcDNA3.1 plasmid derivative. DNA-Lipofectamine complexes were prepared according to the manufacturer´s protocol in serum-free DMEM-F12. Protein expression was followed by Western blotting (Fig. S1B) (also see above) and confocal microscopy (see below).

### (iii) Uptake of amyloid fibers by N2a cells.

To assay the uptake of the *in vitro*-preassembled RepA-WH1(A31V)-Alexa 488-labeled fibers by the N2a cell line, cells were seeded in 35-mm-diameter plates and transiently transfected with pcDNA3.1-*repA-WH1*(*WT*)*-mCherry*, expressing a soluble variant of RepA-WH1 (45, 47), or with pcDNA3.1*-mCherry* as a control (see above and Fig. S1A). Immediately after transfection, N2a cells were exposed to RepA-WH1(A31V)-Alexa 488 fibers (1 μM in equivalent protein monomers) in the growth medium. The next day, cells were extensively rinsed with PBS and further cultured in fresh medium. Fluorescence was subsequently monitored by confocal microscopy 24, 48, and 72 h after the exposure to the fibers.

### (iv) Generation of stable and Tet-ON regulated expression cell lines.

To stably express RepA-WH1(WT)-TagGFP2 and TagGFP2 in the SH-SY5Y cell line, a Tet-ON 3G expression system (Clontech) was used. It was composed of pCMV-*Tet3G* (regulatory) and pTRE3G (response) plasmids. To generate SH-SY5Y cells expressing the transactivator protein (rtTA) upon genomic integration of pCMV-*Tet3G*, 1 μg of this plasmid was transfected into 5 × 10^5^ SH-SY5Y cells with Lipofectamine LTX Plus (1:1 ratio; Invitrogen) and selected for colonies with G418 (0.5 mg/ml). Individual clones, isolated using a cloning cylinder (Sigma), were then screened for luciferase activity (see below). Suitable THN-rtTA/SH-SY5Y clones (i.e., those with high levels of luciferase induction and low levels of basal expression) were subsequently cotransfected with a mixture (10:1) of pTRE3G-*WH1*(*WT*)*-TagGFP2* (or pTRE3G-*TagGFP2*) and a hygromycin linear selection marker. Doubly stable SH-SY5Y transfectants were then selected by screening with hygromycin (0.4 mg/ml) and G418 (0.5 mg/ml) for 2 weeks followed by testing for the best expression of *repA-WH1*(*WT*)*-TagGFP2* and *TagGFP2* genes in the presence/absence of doxycycline (0.5 μg/ml) after 48 h by Western blotting (Fig. S3B; also see above), flow cytometry (Fig. S3C), and confocal microscopy (Fig. S3D) (see below).

### (v) Luciferase assay.

The luciferase assay kit (Promega) was used to test the expression and regulation performance of the selected transactivator-positive clones. Specifically, G418-resistant THN-rtTA/SH-SY5Y clones were seeded in a 6-well culture plate at a density of 5 × 10^5^ cells/well. One day after seeding, the cells were transiently transfected with 5 μg of the pTRE3G-Luc control vector (Clontech) using Lipofectamine LTX (see above). Luciferase expression was induced by adding 0.5 μg/ml doxycycline for 48 h to complete DMEM-F12. In parallel, to test clones for basal luciferase expression, controls with no doxycycline supplied were processed. Afterward, the cells were harvested in RIPA lysis buffer and bioluminescence was monitored at 562 nm using a TD-20/20 Turner Designs luminometer.

### (vi) N2a/SH-SY5Y cocultures.

To explore the cell-to-cell transmissibility of the protein particles, SH-SY5Y stable cells (recipient cells), expressing either soluble WH1(WT)-TagGFP2 or TagGFP2, were seeded on glass coverslips coated with 0.1 mg/ml poly-l-lysine (Sigma) and processed in 24-well plates. At 24 h afterwards, N2a cells (donor cells) transiently transfected with pcDNA3.1-*WH1*(*A31V*)*-mCherry* or pcDNA3.1-*WH1*(*WT*)*-mCherry* were detached with trypsin and transferred to the plates bearing the SH-SY5Y receptor cells (2:1 ratio) and cocultured for 5 days in complete DMEM-F12 with 0.5 μg/ml doxycycline. The cells were then fixed with 4% paraformaldehyde (PFA) during 15 min and stained with DAPI (4′,6-diamidino-2-phenylindole) (Merck-Millipore) (5 min; 1 μg/ml in PBS).

### Flow cytometry.

Flow cytometry was used to monitor fluorescent protein expression in the stable clones for the rtTA transactivator, in either the presence or absence of 0.5 μg/ml doxycycline, at 48 h after induction. Cells were detached by trypsinization from 35-mm-diameter culture dishes and centrifuged at 1,000 rpm for 5 min at 4°C. Then, pellets were resuspended in 2 ml of cold PBS buffer. GFP fluorescence (excitation [Ex], 488 nm; emission [Em], 525 nm) was measured in a Coulter Epics XL (Beckman-Coulter) flow cytometer, analyzing 10,000 cells per sample. Subsequent data analysis was performed using Flowlogic software (v7.2.1).

### Cell proliferation (MTT) assay.

To assess cell proliferation, N2a cells transfected with pcDNA3.1-*WH1*(*Δ*/*WT*/*A31V*/*ΔN37*)*-mCherry*, as described above, were seeded 24 h after transfection into 96-well culture plates at a density of 6 × 10^4^ cells/well. The next day, MTT (Sigma) (0.5 mg/ml in PBS) was supplied and, after 4 h of incubation at 37°C, the MTT was removed and replaced with 200 μl of dimethyl sulfoxide (DMSO). Absorbance was measured at 570 nm in a Varioskan Flash plate reader (Thermo Fisher Scientific). Absorbance values were normalized to those obtained in DMSO.

### Confocal microscopy.

Cells were directly observed at RT by confocal laser microscopy using a Leica TCS-SP5 microscope with a 63× (numerical aperture [NA] = 1.4 to 0.60/oil HCX PL APO) immersion lens objective. The laser lines used for excitation were 514 nm (mCherry), 488 nm (Alexa 488 and TagGFP2), and 405 nm (DAPI and ThS). Images were acquired at 1-μm intervals (Z-sections). Cells were fixed with 4% PFA–PBS for 15 min and rinsed three times in PBS, and staining of nuclei, when required, was performed with 1 μg/ml DAPI solution (Merck-Millipore)–PBS for 10 min, followed by three rinses with PBS. Coverslips were mounted with Fluoromount-G medium (SouthernBiotech) on microscopy slides (Linealab). For amyloid characterization of intracellular particles, transiently transfected N2a cells were grown on coverslips coated with poly-l-lysine for 48 h. Cells were fixed with 4% PFA–PBS for 20 min and rinsed as described above. The cells were then permeabilized with cold 50% methanol (Emsure) in Milli-Q H_2_O for 5 min, incubated with 0.05% thioflavin-S (ThS; Sigma)–12.5% ethanol for 30 min, and rinsed three times in 50% ethanol–distilled H_2_O. Cells were then hydrated for 5 min in PBS and mounted.

### Statistical analysis.

All experiments were carried out in triplicate. GraphPath PRISM (v.6) software was used to estimate the medians and standard deviations of data points and for the analysis of their statistical significance through the use of either Student’s *t* test or one-way analysis of variance (ANOVA).

### Proteomics.

**(i) Gel electrophoresis and trypsin digestion.** The following cell types were studied: (i) N2a cells transfected with pcDNA3.1-*WH1*(*WT*)*-mCherry* or pcDNA3.1-*mCherry* and incubated with *in vitro*-assembled WH1(A31V) fibers for 48 h; (ii) THN-rtTA/SH-SY5Y cells having integrated pTRE3G-*WH1*(*WT*)*-TagGFP2* or pTRE3G*-TagGFP2*, both after induction with Dox (0.5 μg/ml) for 48 h; and (iii) naive N2a and THN-rtTA/SH-SY5Y control cells. Whole-cell extracts (see above) were partially resolved by SDS-PAGE gel (10% polyacrylamide) such that the whole proteome became concentrated in the stacking/resolving gel interface. Protein bands were visualized by colloidal blue staining (Invitrogen), cut into small pieces, and placed in 0.5-ml tubes prior to manual tryptic digestion (71). Excised bands were separately destained with 50 mM ammonium bicarbonate (ABC) and 50% acetonitrile (ACN), dehydrated with ACN, and dried by speed-vacuum centrifugation. Samples were then reduced with 10 mM dithiothreitol (DTT)–25 mM ABC and alkylated with iodoacetamide to reach a final concentration of 50 mM. Then, gel pieces were dried, rehydrated with 12.5 ng/ml porcine trypsin (Thermo Fisher Scientific) in 50 mM ABC, and incubated overnight at 37°C. Peptides were extracted using 100% ACN and 0.5% trifluoroacetic acid, purified using a Zip Tip with 0.6 μl of C_18_ resin (Millipore, Sigma-Aldrich), and dried. Samples were reconstituted in 10 μl of 0.1% formic acid before their analysis by nanosystem liquid chromatography-tandem mass spectrometry (nLC–MS/MS). All peptide separations were carried out on an Easy-nLC 1000 nanosystem (Thermo Fisher Scientific). For each analysis, the sample was loaded into an Acclaim PepMap 100 precolumn (Thermo Fisher Scientific) and eluted through a rapid-separation liquid chromatography (RSLC) PepMap C_18_ column (Thermo Scientific) (50 cm long, 75-μm inner diameter, 2-μm particle size). The mobile phase was 0.1% formic acid–water (solvent A) and 0.1% formic acid–acetonitrile (solvent B). The gradient profile was set, using a flow rate of 300 nl/min, as follows: 5% to 35% solvent B for 100 min, 35% to 45% solvent B for 20 min, 45% to 100% solvent B for 5 min, and 100% solvent B for 15 min. A 4-μl volume of each sample was injected.

**(ii) LC-MS analysis.** MS analysis was performed using a Q-Exactive mass spectrometer (Thermo Scientific). Ionization was performed at a liquid junction voltage of 2,000 and a capillary temperature of 270°C. The full scan method comprised mass selection at *m*/*z* 300 to 1,800, an Orbitrap resolution of 70,000 (at *m*/*z* 200), a target automatic gain control (AGC) value of 3 × 10^6^, and 100 ms of maximum injection time. After the scan, the 15 most intense precursor ions were selected for MS/MS fragmentation. Fragmentation was performed with a normalized collision energy value of 27, and MS/MS scans were acquired with a starting mass of *m*/*z* 200, a 2 × 10^5^ AGC target, a resolution of 17,500 (at *m*/*z* 200), an intensity threshold (IT) of 8 × 10^3^, 2.0 *m*/*z* units as the isolation window, and 100 ms maximum IT. Charge state screening was enabled to reject unassigned ions, singly charged ions, and protonated ions at a level of ≥7. A dynamic exclusion time of 30 s was used to discriminate against previously selected ions.

**(iii) Data processing.** MS data were analyzed with Proteome Discoverer (version 1.4.1.14) (Thermo) using standardized workflows. Mass spectra .raw files were searched against either the Mus musculus Swiss-Prot 2016 database (16,838 protein entries; for the N2a recipient cells) or the Homo sapiens Swiss-Prot 2016 database (20,131 protein entries; for the SH-SY5Y recipient cells), using the Mascot search engine (v.2.6, Matrix Science). Precursor and fragment mass tolerance levels were set to 10 ppm and 0.02 Da, respectively, allowing 2 missed cleavages, carbamidomethylation of cysteines as a fixed modification, and N-terminal methionine oxidation and acetylation as a variable modification. Identified peptides were filtered using the Percolator algorithm with a false-discovery-rate (FDR) (*q*-value) threshold of 0.01 (high-confidence filter settings; FDR < 1%) (72). Proteins contributing peptides detected at least two times (peptide-spectrum match [PSM] value of >1) or having two or more peptides identified were selected for further analysis. Data representing proteins identified in naive N2a murine control cells were subtracted from data representing those found in N2a cells expressing WH1(WT)-mCherry or mCherry and incubated (or not) with fibers. Data representing proteins found in naive human THN-rtTA/SH-SY5Y control cells were subtracted from data representing those found in THN-rtTA/SH-SY5Y cells expressing WH1(WT)-TagGFP2 or TagGFP2 and cocultured (or not) with the murine donor cells. This subtractive approach, by eliminating in the data sets the background of ubiquitous host proteins, facilitated the identification of the relevant proteins specifically present under each set of cocultivation conditions. A Boolean algebra analysis (Venny, v.2.1; https://bioinfogp.cnb.csic.es/tools/venny/) was undertaken to identify proteins exclusively found at, or shared between, the WH1(WT)-mCherry or TagGFP2 recipient cells. The proteins in the subset present in the WH1(WT)-mCherry/TagGFP2 data sets solely upon incubation with the WH1(A31V) fibers/donor cells were then classified according to functional gene ontology (GO) (GeneCards; 73) and cluster analysis (STRING v.11; 74).

### Data accessibility.

Data sets have been submitted to the PRIDE proteomics identification database and are accessible under accession no. PXD018245.
