# Supplementary figures and images for: Intercellular Transmission of a Synthetic Bacterial Cytotoxic Prion-Like Protein in Mammalian Cells
Source: mBio. 2020 Apr 14;11(2):e02937-19. doi: 10.1128/mBio.02937-19 (PMC7157824; doi:10.1128/mBio.02937-19)

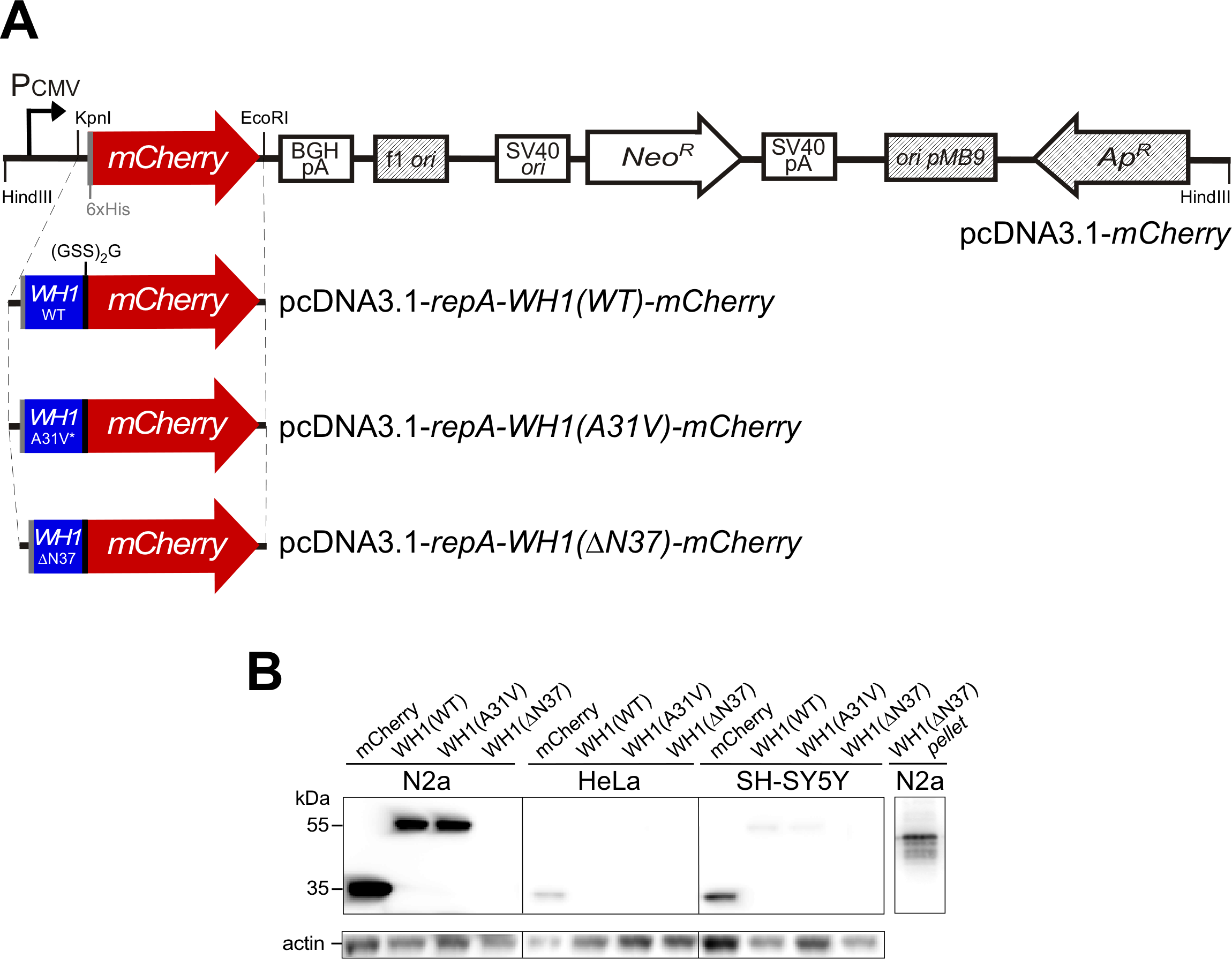

Supplement: FIG S1 [file mBio.02937-19-sf001.tif]

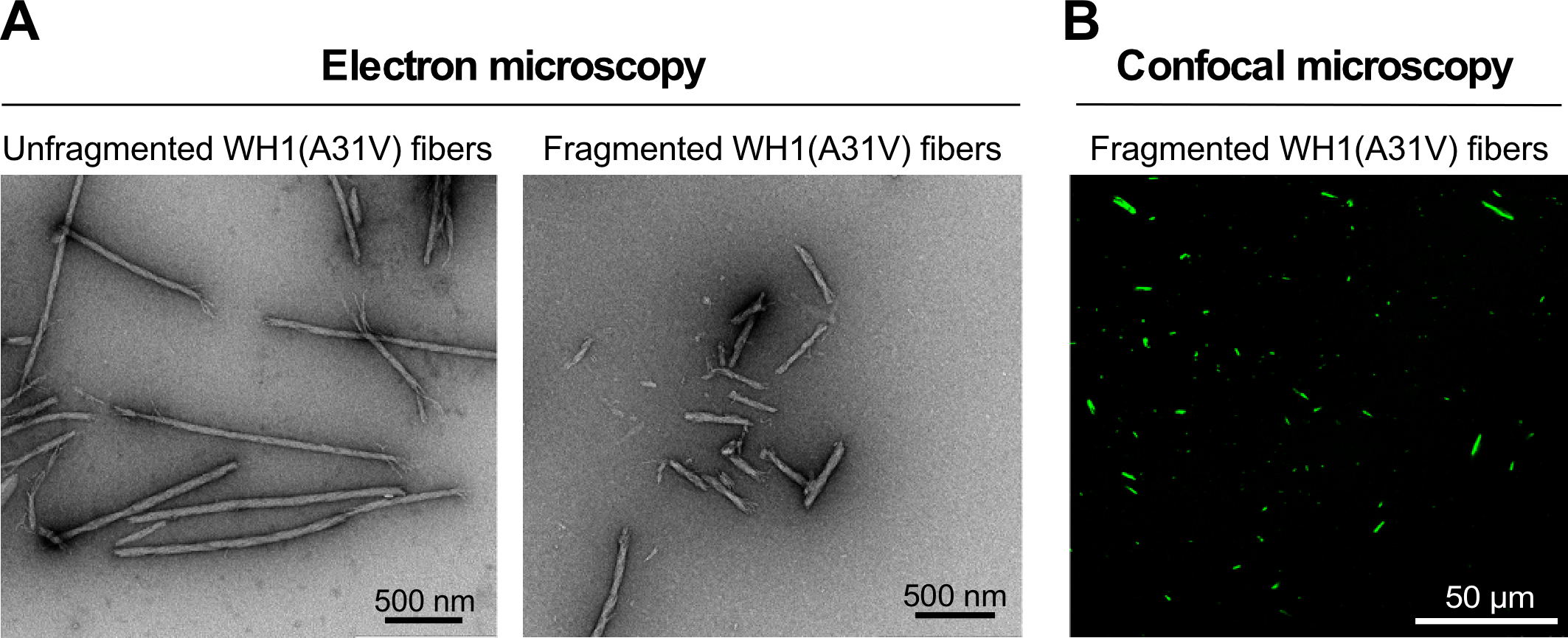

Supplement: FIG S2 [file mBio.02937-19-sf002.tif]

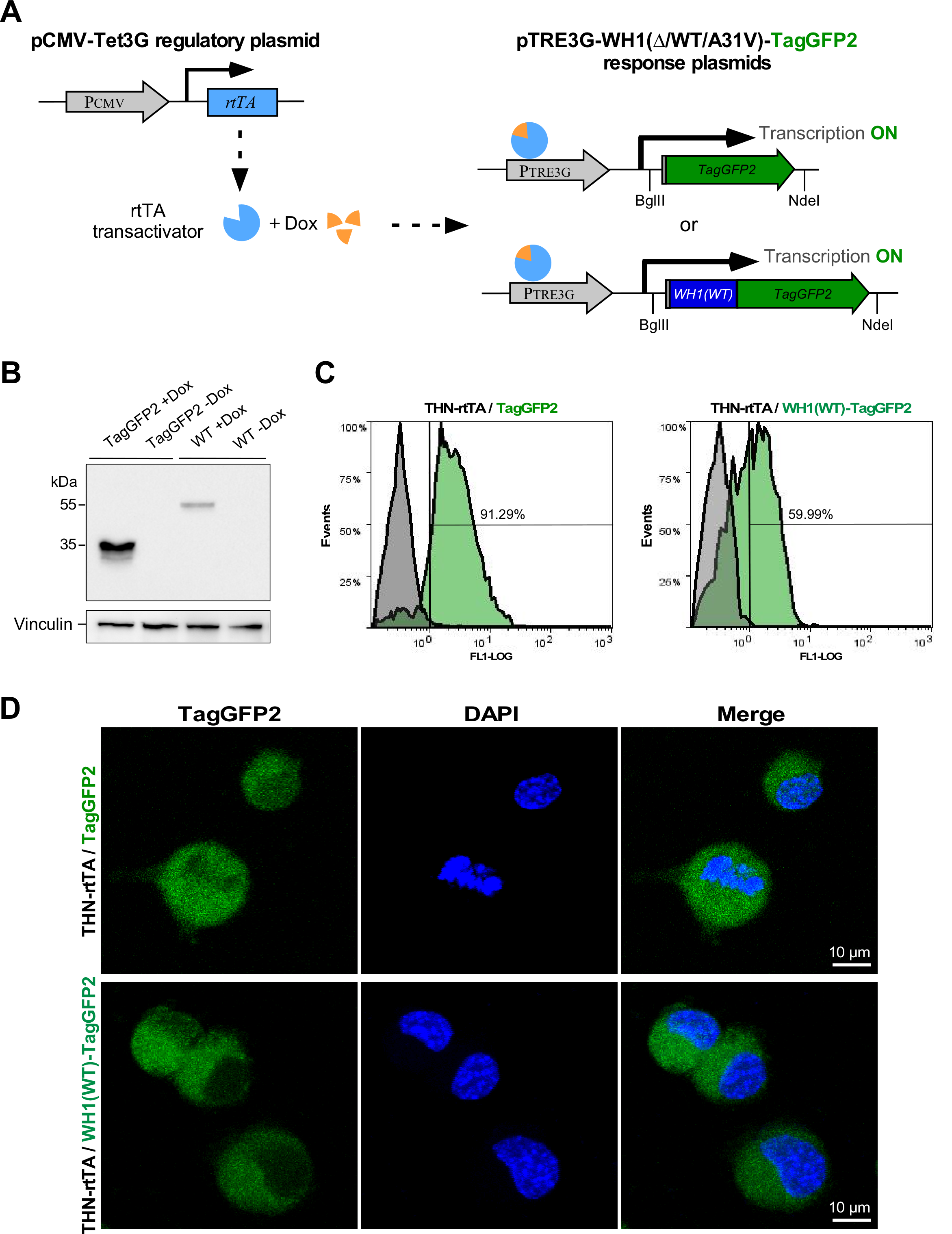

Supplement: FIG S3 [file mBio.02937-19-sf003.tif]

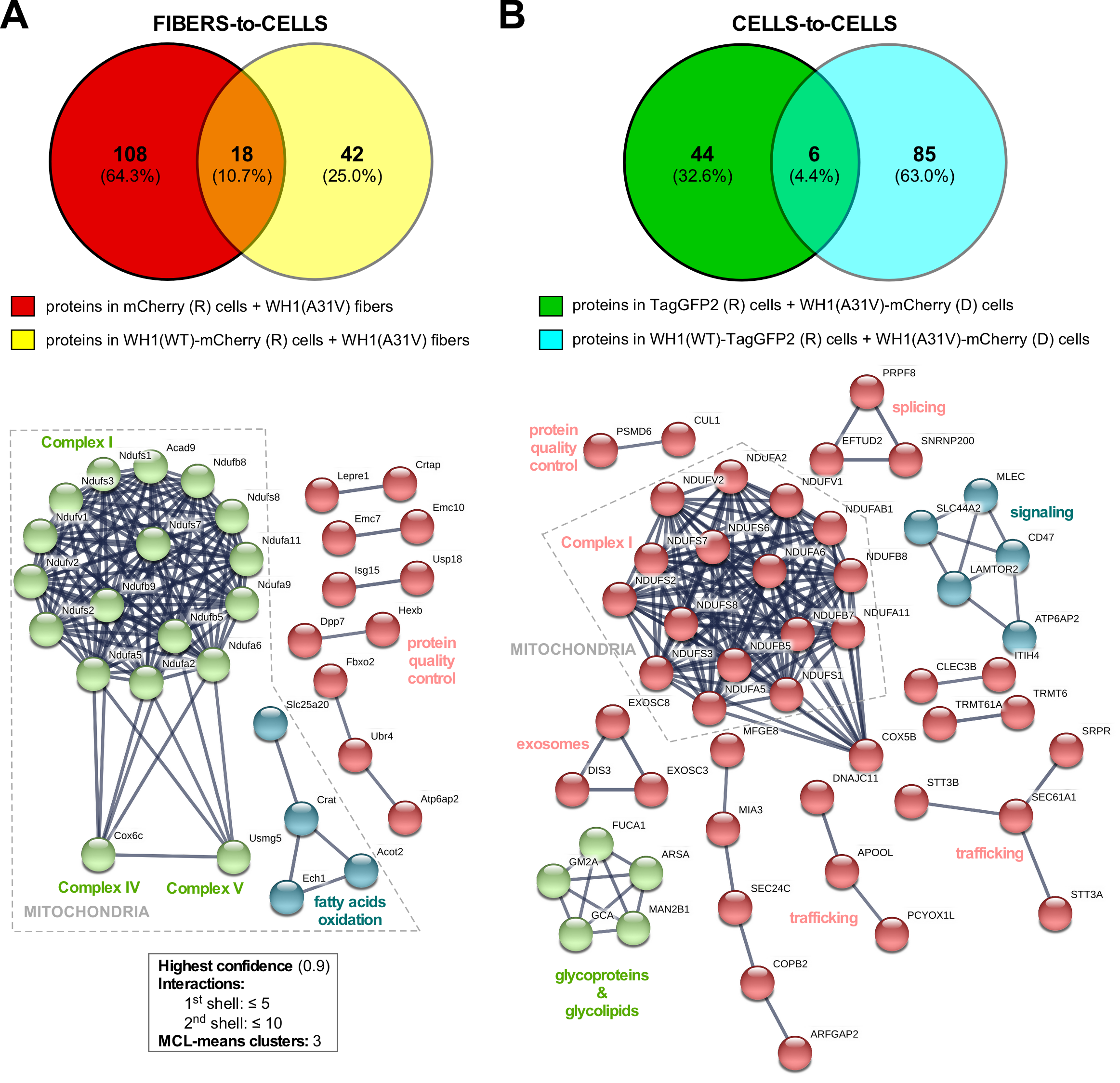

Supplement: FIG S4 [file mBio.02937-19-sf004.tif]
